# Sexual violence and rape among young migrants in Sweden: a cross-sectional study on prevalence, determinants, perpetrators, and reporting patterns

**DOI:** 10.3389/fpubh.2024.1471471

**Published:** 2024-11-21

**Authors:** Mazen Baroudi, Faustine Kyungu Nkulu Kalengayi

**Affiliations:** Department of Epidemiology and Global Health, Umeå University, Umeå, Sweden

**Keywords:** sexual violence, rape, migrants, young people, LGBTQ, non-binary, sexual rights, Sweden

## Abstract

**Background:**

Young migrants are particularly vulnerable to SV (SV) due to their age and the challenges of migration. However, there is limited knowledge regarding SV among young migrants in Sweden. This study aims to assess the prevalence, determinants, perpetrators, and reporting patterns of SV and rape.

**Methods:**

We analyzed data from the 2018 survey on migrants’ sexual and reproductive health and rights, involving 1773 migrants aged 16–29. We estimated prevalence rates and calculated crude and adjusted prevalence ratios (APR) with 95% confidence intervals (CI) using descriptive and log-binomial regression analyses.

**Results:**

The overall prevalence of SV was 25.1%, with rape at 9%. Higher SV prevalence were reported by non-binary individuals (APR: 2.60, CI: 1.54 to 4.38), Lesbian, Gay, Bisexual and Asexual (LGBA) individuals (APR: 1.56, CI: 1.22 to 2.01), those with 10–12 years (APR: 1.35, CI: 1.04 to 1.74) and over 12 years of education (APR: 1.61, CI: 1.23 to 2.11), and migrants awaiting asylum decisions (APR: 1.67, CI: 1.25 to 2.23). Rape prevalence was higher among those born in non-conflict settings (APR: 2.38, CI: 1.43 to 3.97), non-binary individuals (APR: 3.32, CI: 1.35 to 8.18), and LGBA individuals (APR: 1.68, CI: 1.02 to 2.75). Although men reported higher SV and rape levels than women in descriptive and bivariate analyses, these differences were not significant in multivariate analyses. Perpetrators included strangers (46.3%), partners (24.8%), family/friends (15.8%), and colleagues (15.4%). Most survivors did not report SV (63.7%) but confided in friends/relatives (29%) and trusted individuals like teachers/counselors (9.5%), with fewer reporting to authorities (3.4%).

**Conclusion:**

The findings urge policymakers to prioritize targeted interventions, raise awareness, provide comprehensive support services tailored to the diverse needs of migrant groups, various perpetrator types, and the individual, systemic, and structural factors influencing reporting behaviors. These initiatives should adopt a migration-trajectory approach that recognize that migrants may have experienced SV throughout their journey and consider the unique experiences and vulnerabilities of non-binary individuals, LGBA individuals, those with low education levels, and migrants without formal residence status, regardless of their origin.

## 1 Introduction

In various regions of the world, international migration has emerged as a significant driver of population dynamics. Notably, for high-income countries including Sweden during the period from 2000 to 2020, the impact of international migration on population growth surpassed the natural balance of births and deaths (1). Despite growing global attention to human mobility, young migrants remain a distinct group whose specific needs, rights, and challenges are inadequately addressed in broader political discussions and national policies. There is limited research and data focused on understanding the impact of migration on young people, even though they constitute a substantial proportion of all international migrants (2). Among the latest estimates, approximately 11% of the 281 million international migrants in 2020 were aged 15–24 years (3). However, it is crucial to disaggregate data on young migrants by age, gender, and whether they are accompanied by parents, family members, guardians, or sponsors. This information is crucial for evaluating vulnerability levels and identifying protection needs during transit and at destinations (3). For instance, during the 2015 ‘refugee crisis,’ Sweden received the highest share (37%) of unaccompanied minor applications, totaling 34,295, while in 2016, Germany had the highest share (60%) with 35,935 applications (3). In 2018, approximately one-fifth of Sweden’s population was foreign-born (19%), and among young people aged 16–29 years, around 21% were foreign-born (4).

Currently, there is no universally agreed-upon definition of ‘youth’ or ‘international migrant.’ This lack of consensus complicates the collection and analysis of disaggregated data across origin, transit, and destination countries, hindering the development of targeted policies for young people (1, 2). For statistical purposes, the United Nations (UN) defines youth as ‘those persons between the ages of 15 and 24 years,’ while acknowledging that Member States may have other definitions (3). Meanwhile, the UN Statistical Commission provides a specific definition of ‘migrant’ based on foreign-born status, but analysts sometimes use alternative definitions for analytical purposes (1). In legal, administrative, research, and statistical contexts, various specific definitions are employed for migration-related terms including in relation to place of birth, citizenship, place of residence and duration of stay. Various widely accepted definitions have been developed, including those outlined in UN Department of Economic and Social Affair’s 1998 Recommendations on Statistics of International Migration [e.g., individuals living outside their country of birth for more than 12 months (1)]. The International Organization for Migration defines ‘migrants’ as individuals who move away from their usual country of residence, either temporarily or permanently, for diverse reasons and settle in another country (5). These broad definitions encompass people from different countries, socio-cultural backgrounds, legal statuses, durations of stay, and age groups, resulting in varying levels of vulnerability and access to services. Additionally, governments’ definitions and categories used during data collection at border entry and exit points and throughout the asylum process also exhibit variation (3).

Statistics Sweden (*Statistikmyndigheten SCB*) defines ‘(im)migrants’ (*Invandrare*) as individuals who move to Sweden and are registered in the Swedish population register. To be registered, one must intend and have the right to stay in Sweden for at least 12 months. The rules for registration differ among migrant groups: Nordic citizens can freely immigrate to Sweden, while EU citizens outside the Nordic countries must apply for the ‘right of residence’ to stay longer than 3 months. Citizens of other countries require a residence permit issued by the Swedish Migration Agency (6). On the other hand, the Swedish Agency for Youth and Civil Society (*Myndigheten för Ungdom och Civilsammhällesfrågor:MUCF*) primarily targets youths and young adults aged between 13 and 25 years but its policies can also apply to other age groups (7). The Public Health Agency of Sweden identifies “youths and young adults,” defined as individuals aged 15–29 years, as a priority group in the National Strategy for Sexual and Reproductive Health and Rights (SRHR). This strategy emphasizes the need to strengthen the health and rights of this group (8). In this study, young migrants are defined as individuals aged 16–29 years who were born outside of Sweden, irrespective of their legal status, reasons for migrating, or the length of time they have lived in Sweden.

Migration itself is not inherently a risk. Instead, the conditions surrounding the migration process significantly influences the general health and SRHR of migrants, their families, and communities, acting as a social determinant of health. It intersects with existing determinants and introduces an additional layer, thereby increasing health risks and negative outcomes. This process also contributes to widening health inequalities between non-migrants and migrants, as well as among different migrant groups (9). While evidence suggests that most migrants begin their journeys as young, fit, and healthy individuals, the migration experience can expose certain groups to multiple vulnerabilities and (health) risks at all levels of the socio-ecological model (9). Notably, evidence highlights that the migration context heightens vulnerability to various forms of violence, including sexual violence (SV) (10). The World Health Organization (WHO) defines SV as any coercive act directed against a person’s sexuality, regardless of the perpetrator’s relationship to the victim. This encompasses rape, which involves forced or coerced penetration of the vulva or anus using a penis, other body part, or object regardless of the gender of the perpetrator (11). Throughout the migratory process—before departure (in the country of origin), during transit, upon arrival in the destination country, and even upon return—migrants can be subjected to SV, abuse, exploitation, and harassment, including rape. Despite that, research on SV in migrant populations is much more limited compared to the general population (10, 12–14).

Existing evidence indicates that SV is more prevalent among migrants compared to the general population (12–14). A review of the global literature on SV among migrants reveals a wide range of prevalence rates, from 0 to 99.8%. While reports of SV in migrants are documented on all continents, about four in ten (42%) of the included papers focused on refugees from Africa, with prevalence rates ranging from 1.3 to 100%. Rape emerges as the most frequently reported form of SV, appearing in 65% of studies, with prevalence rates ranging from 0 to 90.9% (13–15). Evidence from Europe shows similar patterns, with the prevalence of SV among migrants varying from 22 to 69% across different studies and countries. However, most of these studies focused on specific groups, such as migrants, applicants for international protection, refugees (MARs), asylum seekers, unaccompanied children and undocumented or irregular migrants (12, 15). Although women and girls are disproportionately affected, all genders remain vulnerable (12–14). Regarding perpetrators, a reverse pattern is observed in the literature. For instance, in one study conducted in Belgium and the Netherlands, most perpetrators were men (73%), only 6% were women, 19.6% had an unspecified gender, and 1.5% were transgender (10, 12). Interestingly, another study found an equal number of male and female perpetrators (16). The perpetrators of SV are often either unknown to the victims (e,g, strangers) or have some form of relationship with them (partner, friend or relative) (10, 12–14).

SV constitutes a significant public health challenge among migrants, with far-reaching consequences across various dimensions, including negative impacts on socio-economic well-being, physical health, mental health, sexual health, and reproductive health. These consequences are exacerbated by the fear of disclosure and the associated stigma that prevent survivors from disclosing (10, 13, 16, 17). Unfortunately, most survivors do not seek formal assistance or report to the police, likely due to the fear of disclosure, societal stigma, and complex barriers that hinder access to available information and services (13, 16, 18). Instead, survivors tend to confide in informal networks, such as friends, relatives, parents, partners, and acquaintances (16).

Nevertheless, migrants form a heterogeneous group, originating from diverse countries, spanning different age groups, gender identities, sexual orientation, reasons for migration, legal statuses, and migration experiences. These factors significantly influence their vulnerability to SV with certain subgroups such as young migrants, facing heightened vulnerability than others (3, 9). Both young age and migration experience contribute to this vulnerability, affecting their exposure to SV and overall sexual and reproductive health outcomes (17, 19). Young migrants are particularly susceptible to various forms of violence, exploitation, and rights violations. This vulnerability is further exacerbated for those migrating without parental or guardian support or those who deviate from societal norms related to ethnicity, gender, gender identity, expression, disability, and sexual orientation (17). Despite these multiple dimensions of vulnerability, research on SV among young migrants in Europe, including Sweden, remains scarce (15, 18). A descriptive analysis of data from the migrants’ sexual and reproductive health and rights (MSRHR-2018) survey revealed that approximately 25% of migrant respondents aged 16–29 years reported experiencing SV. Disparities in prevalence were observed based on gender, level of education, and region of origin (20). However, these findings remained descriptive, and no significant associations could be identified between SV and socio-demographic characteristics. Thus, this study aims to assess the prevalence, determinants, perpetrators and reporting patterns of SV and rape.

## 2 Materials and methods

### 2.1 Study design, setting and survey instrument

We analyzed data from the 2018 Migrants’ Sexual and Reproductive Health and Rights (MSRHR-18) survey. The MSRHR-18 survey was developed using questionnaires from national surveys such as UngKab15 (21), Sexual and Reproductive Health and Rights in Sweden 2017 (SRHR-2017) (22), and the British National Survey of Sexual Attitudes and Lifestyles (Natsal-3) (23). The complete MSRHR-18 survey is available here (24). The questionnaire was initially designed in Swedish and English, then linguistically and culturally adapted into Arabic, Dari, Somali, and Tigrinya, the languages spoken by the largest migrant groups in Sweden. It included 71 questions addressing a wide range of topics, including general health, safety, social relationships, sexual and reproductive health and rights, and socio-demographic characteristics. In alignment with the study’s objectives, the dataset for this study exclusively focused on questions related to SV and socio-demographic characteristics.

The Swedish National Strategy for Sexual and Reproductive Health and Rights (SRHR) identifies six priority groups, including youths and young adults (15–29 years) people and individuals with migration experience. These groups face worse sexual and reproductive health outcomes compared to the general population, and their rights are often overlooked. To achieve equitable SRHR for the entire population, it is essential to strengthen support and interventions for these groups (8). The recently launched National Action Plan (NAP) for SRHR (2023–2033) also emphasizes advancing knowledge and implementing targeted actions specifically tailored to address SRHR issues within these priority groups (25). Against this background, the Public Health Agency of Sweden (*Folkhälsomyndigheten*) commissioned the MSRHR-2018 survey to address the underrepresentation of migrants in national surveys, following the National SRHR-2017 survey. This study targeted young migrants aged 16 to 29 residing in various parts of Sweden, with a particular focus on those from low-and middle-income countries who had moved to Sweden within the past 10 years.

### 2.2 Data collection

**Figure 1:**
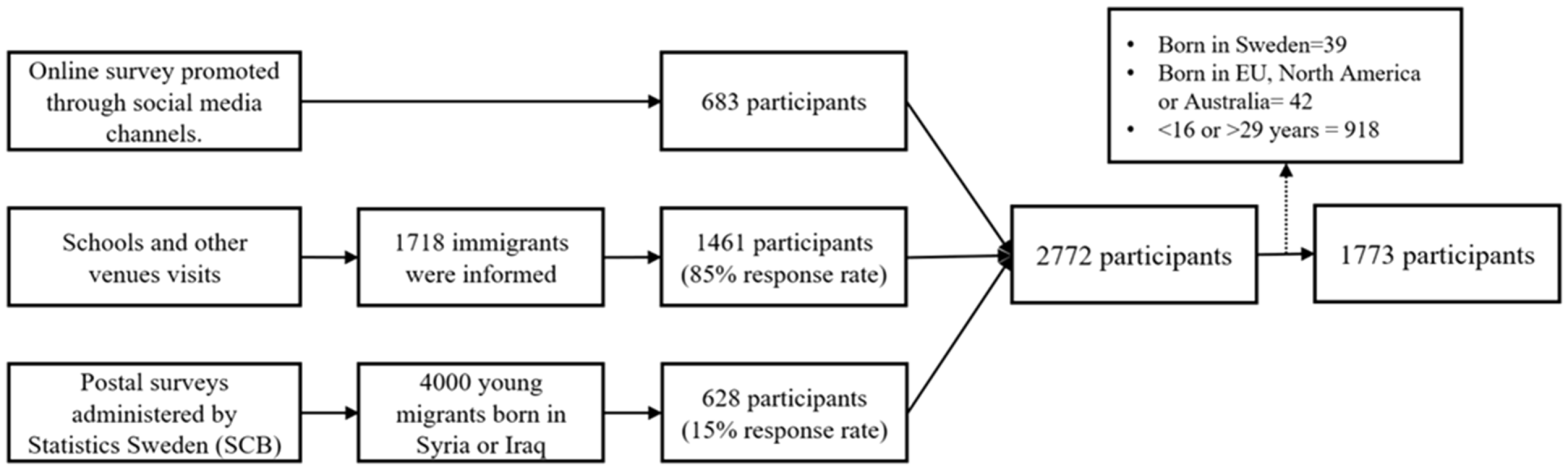
Data collection modes and study participants.

The survey was conducted from March to September 2018 through school visits, mail distribution by Statistics Sweden (SCB), and online platforms. We used language schools as venues where the target population is likely to be found. With permission from school authorities, we administered questionnaires in schools or sent them via mail. Convenience sampling was employed, as a practical approach for reaching migrants, who are often considered hard-to-reach groups. All students present at participating schools on the survey day were included, with bilingual assistants facilitating participation of respondents with limited Swedish language skills. This was supplemented by a postal survey, sent by SCB, targeting migrants from Syria and Iraq due to budget restrictions. Additionally, the online survey was promoted through social media channels. Written informed consent was obtained from all participants after providing detailed information about the study, emphasizing voluntary participation, confidentiality of responses, and anonymity of reporting. Out of 2,772 migrant respondents, 1,773 were included in the analysis after excluding those born in Sweden, other high-income countries, and participants younger than 16 or older than 29 years. Figure 1 provides detailed information about the data collection methods and study participants.

### 2.3 Measures

#### 2.3.1 Outcome variables

The study’s primary outcomes were SV and rape. We adhered to the World Health Organization’s (WHO) definition of SV, which encompasses any sexual act (whether verbal, physical, or involving internet harassment) committed against a person’s will. Participants were asked whether they had ever experienced any form of sexual act against their will. Rape was specifically defined as oral, vaginal, or anal intercourse forced upon an individual against their consent (11). These two variables were dichotomized as ‘Yes’ for those who have ever experienced and ‘No’ for those who have never experienced.

#### 2.3.2 Independent variables

Independent variables included country of birth, age, gender, sexual orientation, level of education, residency status, and reason for migration. Country of birth was classified into conflict and non-conflict settings based on the 2018 war report published by the Geneva Academy (26). Age was divided into three categories: teenagers (16–19 years), youths (20–25 years), and young adults (26–29 years). Gender was categorized as man, woman, and non-binary. Sexual orientation was grouped into three categories: heterosexual; lesbian, gay, bisexual, asexual, and other (LGBA+); and prefer not to answer. Education level was grouped into primary or less (≤ 9 years), secondary (10–12 years), and tertiary (> 12 years). Residency status included: awaiting a decision on a residence permit in Sweden, holding a residence permit since 2016 or later, and holding a residence permit before 2016. Reasons for migration were categorized as seeking asylum, family reunification, work, and other reasons.

#### 2.3.3 Other variables

The study also examined other variables related to SV. The perpetrators were categorized into different groups: partners (individuals with whom the victim has been in a relationship), family members or friends, colleagues or classmates, strangers, and others (such as teachers, bosses, or supervisors). Participants were also asked whether they talked about or reported their experiences (Yes/No) and to whom they reported. The recipients of these reports were classified as follows: None, Authority figures (social services or police), friends or relatives, and others (including bosses, employers, teachers, or school counselors).

### 2.4 Data analysis

We conducted a descriptive analysis to characterize the sample, assess the prevalence of SV and rape, and examine the perpetrators and reporting patterns. Subsequently, we employed log-binomial regression to calculate both crude and adjusted prevalence ratios for SV and rape, aiming to identify their determinants. The regression model was adjusted for various factors, including country of birth, age, gender, sexual orientation, education, resident permit status, and reason for migration. Our prevalence estimates are reported with 95% confidence intervals, and statistical significance was determined by a *p* value below 0.05. The analysis was performed using Stata 15 software.

### 2.5 Ethical considerations

The Research Ethics Committee at Umeå University issued an ethical approval for the study [Dnr 2017/515–31]. Participants provided their written consent following a comprehensive briefing about the research. They were assured that their involvement was entirely optional, they had the freedom to exit the study whenever they wished without any consequences, their individual answers would remain private, and the findings would be presented without revealing their identities.

## 3 Results

### 3.1 Sample characteristics

The sample consisted of 1,773 participants. The majority were born in conflict settings (90%) and migrated to Sweden to seek asylum (72%). Slightly over half (56%) received a resident permit less than 2 years before the survey. Approximately 12% were still awaiting a decision on their asylum application. Among the participants, 63% identified as men, 73% were heterosexual, 45% attended school for 9 years or less, and nearly four in ten (38%) were teenagers aged 16 to 19 years. Detailed sample characteristics are provided in Table 1.

**Table 1:** Sample characteristics.

	Number	Percentage
All	1773	100.00
Country of birth		
Conflict	1,565	90.83
No conflict	158	9.17
Age group		
Teenagers	670	37.79
Youths	592	33.39
Young adults	511	28.82
Gender identity		
Woman	586	34.88
Man	1,06	63.10
Non-binary	34	2.02
Sexual orientation		
Heterosexual	1,134	73.07
LGBA	201	12.95
Do not want to answer	217	13.98
Education		
<=9 years	751	44.86
10–12 years	482	28.79
>12 years	441	26.34
Residence permit		
Still waiting	188	11.56
2015 or earlier	528	32.45
2016 or later	911	55.99
Reason of migration		
Asylum	1,108	72.23
Family reunion	313	20.40
Other	113	7.37

### 3.2 Prevalence of sexual violence and rape

Overall, approximately 25% of participants reported victimization related to SV, with around 9% experiencing rape. The highest prevalence of SV was observed among migrants from non-conflict areas (31%), teenagers (26%) and youths (27%), non-binary individuals (45%), followed by men (26%), LBGA+ individuals (37%), and those with more than 12 years of education (29%). Additionally, participants awaiting a decision on their resident permit reported a significantly higher prevalence of SV (40%) compared to their counterparts (see Table 2).

**Table 2:** Prevalence of sexual violence and rape by individual characteristics.

	Sexual violence	Rape
	Number (%)	Number (%)
All	400 (25.09)	143 (8.97)
Country of birth		
Conflict	347 (24.52)	114 (8.06)
No conflict	45 (31.47)	24 (16.78)
Age		
Teenagers (16–19 years)	155 (26.32)	63 (10.70)
Youth (20–25 years)	146 (26.94)	47 (8.67)
Young adult (26–29 years)	99 (21.38)	33 (7.13)
Gender		
Woman	111 (20.67)	34 (6.33)
Man	248 (26.22)	95 (10.04)
Non-binary	13 (44.83)	5 (17.24)
Sexual orientation		
Heterosexual	258 (24.25)	89 (8.36)
LGBA	68 (36.76)	26 (14.05)
Do not want to answer	39 (19.50)	17 (8.50)
Education		
<=9 years	148 (22.32)	66 (9.95)
10–12 years	119 (27.05)	45 (10.23)
>12 years	122 (28.77)	26 (6.13)
Residence permit		
Still waiting	65 (39.88)	25 (15.34)
2015 or earlier	105 (21.38)	39 (7.94)
2016 or later	188 (23.01)	62 (7.59)
Reason of migration		
Asylum	268 (26.43)	91 (8.97)
Family reunion	58 (21.09)	23 (8.36)
Other	28 (28.57)	12 (12.24)

Regarding rape, the highest prevalence was reported by migrants from non-conflict areas (17%), teenagers (11%), non-binary individuals (17%) followed by men (10%), LBGA+ individuals (14%), and those with less than 12 years of education (10%). Similarly, participants waiting for a resident permit reported a higher prevalence of rape (15%) compared to their respective counterparts (see Table 2).

### 3.3 Factors associated with exposure to sexual violence and rape

The bivariate analyses revealed significant associations between the experience of SV and various factors, including gender, sexual orientation, education, and the year of the decision on residence permits. In the multivariate analysis, most of these associations remained statistically significant. Compared to the reference groups, non-binary individuals experienced significantly higher rates of SV (Adjusted Prevalence Ratio [APR]: 2.60; 95% Confidence Interval [CI]: 1.54 to 4.38). LGBA individuals also showed a higher prevalence (APR: 1.56; CI: 1.22 to 2.01). Participants with 10 to 12 years of education (APR: 1.35; CI: 1.04 to 1.74) and those with more than 12 years of education (APR: 1.61; CI: 1.23 to 2.11) faced a higher risk as well. Additionally, participants still awaiting a resident permit exhibited a higher prevalence (APR: 1.67; CI: 1.25 to 2.23). Notably, there was no statistically significant difference in experience of SV between men and women or between migrants born in conflict areas and those born in non-conflict areas.

Regarding experience of rape, the crude results showed associations with country of birth, age, gender, sexual orientation, education, and resident permit. In the multivariate model, the association between exposure to rape and country of birth, gender, and sexual orientation remained significant. Specifically, migrants born in non-conflict areas (APR: 2.38; CI: 1.39 to 3.12), non-binary individuals (APR: 3.32; CI: 1.35 to 8.18), and LGBA individuals (APR: 1.68; CI: 1.02 to 2.75) had a higher prevalence of rape compared to their reference groups (see Table 3).

**Table 3:** Crude and adjusted prevalence ratios of sexual violence and sexual assault.

	Sexual violence		Rape	
	Crude PR (CI)	Adjusted PR (CI)	Crude PR (CI)	Adjusted PR (CI)
Country of birth				
Conflict	ref	ref	ref	ref
No conflict	1.28 (0.99–1.66)	1.11 (0.79–1.56)	2.08 (1.39–3.12)	2.38 (1.43–3.97)
Age group				
16–19 years	ref	ref	ref	ref
20–25 years	1.02 (0.84–1.24)	0.98 (0.75–1.28)	0.81 (0.57–1.16)	1.02 (0.62–1.67)
26–29 years	0.81 (0.65–1.01)	0.82 (0.61–1.11)	0.67 (0.45–1.00)	0.99 (0.57–1.73)
Gender identity				
Woman	ref	ref	ref	ref
Man	1.27 (1.04–1.54)	1.10 (0.87–1.40)	1.59 (1.09–2.31)	1.27 (0.81–2.01)
Non-binary	2.17 (1.40–3.36)	2.60 (1.54–4.38)	2.72 (1.15–6.44)	3.32 (1.35–8.18)
Sexual orientation				
Heterosexual	ref	ref	ref	ref
LGBA	1.52 (1.22–1.88)	1.56 (1.22–2.01)	1.68 (1.12–2,53)	1.68 (1.02–2.75)
Do not want to answer	0.80 (0.60–1.09)	0.78 (0.54–1.12)	1.02 (0.62–1.67)	0.79 (0.41–1.52)
Education				
<=9 years	ref	ref	ref	ref
10–12 years	1.21 (0.98–1.49)	1.35 (1.04–1.74)	1.03 (0.72–1.47)	1.15 (0.74–1.77)
>12 years	1.29 (1.05–1.58)	1.61 (1.23–2.11)	0.62 (0.40–0.95)	0.65 (0.36–1.16)
Residence permit				
2016 or later	ref	ref	ref	ref
Still waiting	1.73 (1.38–2.17)	1.67 (1.25–2.23)	2.02 (1.31 to 3.12)	1.61 (0.93–2.79)
2015 or earlier	0.93 (0.75 to 1.15)	0.92 (0.73–1.17)	1.05 (0.71–1.54)	1.14 (0.73–1.79)
Reason of migration				
Asylum	ref	ref	ref	ref
Family reunion	0.80 (0.62–1.03)	0.89 (0.66–1.19)	0.93 (0.6–1.44)	0.74 (0.41–1.32)
Other	1.08 (0.78–1.50)	0.99 (0.65–1.51)	1.36 (0.78–2.4)	0.78 (0.33–1.84)

### 3.4 Reported perpetrators

The most reported perpetrators, in descending order, were strangers (46%), partners (25%), family members or friends (16%), colleagues (15%), and other individuals (11%). Migrants born in non-conflict areas reported partners (42%) and strangers (79%) as perpetrators to a higher degree than those born in conflict areas (22 and 50% respectively). Women (31%) reported partners as perpetrators more frequently than men (23%) and non-binary individuals (18%), while men (56%) and non-binary individuals (55%) reported strangers as perpetrators more often than women (47%). LGBA+ individuals reported both strangers (57%) and partners (29%) as perpetrators to a greater extent than heterosexuals (50 and 24% respectively). Participants with 9 years of education or less reported strangers (63%) as perpetrators more frequently than those with 10–12 years of education (42%) or more (52%). Additionally, participants who received their residence permit in 2015 or earlier reported strangers (59%) and partners (30%) as perpetrators more often than those who received their permit later (47 and 21% respectively) or were still awaiting a decision for their application (54 and 22% respectively). Lastly, participants who moved for family reunions reported strangers and partners as perpetrators more frequently than others (see Table 4).

**Table 4:** The perpetrators by individual characteristics.

	Stranger	Partner	Family/friend	Colleague	Others
	%	%	%	%	%
All	46.30	24.76	15.76	15.43	10.93
Country of birth					
Conflict	49.62	22.18	15.04	15.04	10.90
No conflict	78.95	42.11	21.05	13.16	10.53
Age group					
Teenagers (16–19 years)	59.69	28.68	13.95	17.83	13.18
Youths (20–25 years)	48.60	17.76	17.76	11.21	10.28
Young adults (26–29 years)	50.67	28.00	16.00	17.33	8.00
Gender identity					
Woman	46.67	31.11	15.56	10.00	7.78
Man	56.15	22.46	16.04	18.72	10.16
Non-binary	54.55	18.18	18.18	27.27	18.18
Sexual orientation					
Heterosexual	50.52	24.48	13.02	16.15	9.38
LGBA	56.92	29.23	18.46	13.85	12.31
Do not want to answer	59.26	25.93	22.22	18.52	11.11
Education					
<=9 years	62.71	27.97	16.95	17.80	11.86
10–12 years	52.08	28.13	11.46	15.63	8.33
>12 years	42.22	17.78	17.78	11.11	13.33
Residence permit					
Still waiting	54.24	22.03	16.95	13.56	13.56
2015 or earlier	59.04	30.12	14.46	14.46	15.66
2016 or later	46.72	21.17	13.87	17.52	5.84
Reason of migration					
Asylum	52.36	23.11	14.62	17.45	10.85
Family reunion	58.54	36.59	12.20	12.20	4.88
Other	45.45	13.64	13.64	9.09	9.09

### 3.5 Reporting patterns

Among participants exposed to SV, the majority (64%) did not disclose or report the incidents to anyone. Approximately one-fourth confided in a friend and/or a relative (29%), while only around 3% reported the incidents to the police or social services. Notably, none of the non-binary individuals reported the incidents to the authorities. Women shared their experiences (38%) more frequently with friends and/or relatives compared to men (27%) and non-binary individuals (17%). Conversely, non-binary individuals were more reticent, with 75% choosing not to talk or report the incidents to anyone, surpassing both men (65%) and women (57%). Participants still awaiting a decision had the highest prevalence (69%) of non-reporting. For a detailed breakdown of reporting patterns based on individual characteristics (see Table 5).

**Table 5:** Reporting patterns of sexual violence by individual characteristics.

	No one	Authority	Friend/relatives	Other
	%	%	%	%
All	63.72	3.35	28.96	9.45
Country of birth				
Conflict	63.70	3.56	28.83	9.61
No conflict	62.50	2.50	30.00	10.00
Age group				
Teenagers (16–19 years)	59.85	3.65	27.74	16.06
Youths (20–25 years)	67.27	2.73	29.09	3.64
Young adults (26–29 years)	65.43	3.70	30.86	6.17
Gender identity				
Woman	56.67	4.44	37.78	8.89
Man	64.71	3.43	27.45	9.31
Non-binary	75.00	0.00	16.67	8.33
Sexual orientation				
Heterosexual	62.25	3.43	31.86	7.84
LGBA	63.64	4.55	25.76	13.64
Do not want to answer	71.88	0.00	18.75	9.38
Education				
<=9 years	62.70	5.56	30.16	10.32
10–12 years	68.37	2.04	23.47	9.18
>12 years	60.42	2.08	33.33	8.33
Residence permit				
Still waiting	69.35	1.61	19.35	12.90
2015 or earlier	60.71	4.76	34.52	8.33
2016 or later	59.86	2.72	33.33	9.52
Reason of migration				
Asylum	64.73	3.13	27.23	10.71
Family reunion	66.67	2.22	28.89	4.44
Other	60.87	4.35	30.43	13.04

## 4 Discussion

This study reveals that 25% of participants experienced SV, with 9% reporting incidents of rape. Notably, prevalence rates varied across socio-demographic groups. Participants who identified with gender identities other than ‘man’ or ‘woman,’ as well as LGBA individuals and those who attended school for 10 years or more, reported SV more frequently than their counterparts. Although men reported higher levels of SV and rape than women in descriptive and bivariate analyses, these differences were not significant in multivariate analyses. Among those born in non-conflict settings, the prevalence of rape was over 2 times higher than among those born in conflict settings. Additionally, individuals with non-binary gender identities experienced over 3 times higher rates of rape compared to women. LGBA individuals also faced a higher risk of SV compared to heterosexual individuals. Commonly mentioned perpetrators were strangers, followed by partners, family members/friends, and colleagues. The study further highlights that survivors often do not report incidents or confide in anyone about their experiences. When they do share, it is typically with friends, relatives, teachers, or counselors. Reporting to authorities such as the police or social services remains less common. However, there were some disparities in reporting patterns.

### 4.1 Sexual violence and rape are prevalent among young migrants

This study sheds light on the prevalence of SV victimization among young migrants in Sweden, highlighting their vulnerability. Previous research indicates that young migrants face increased risks of experiencing SV at all stages of the migration process (13–15). Our findings reveal that SV rates among this group are comparable to national and international studies. These include the 26% rate of sexual harassment and 24% rate of unwelcome touching reported in a national survey of the general population (22), the 26% weighted incidence of SV among recently arrived asylum seekers in France (27), and the 27.6% rate of sexual assault reported by Doctors of the World (*Médecins du Monde, MdM*) in their 2014 report on access to healthcare for vulnerable people, such as migrants, asylum seekers and refugees (MARs), in 11 European countries, including Sweden (28). However, the SV rate among young women in our study (21%) is markedly lower than the 57% reported for young women in the same age (16–29 years) group in the same national survey (22). This contrast is even more pronounced when compared to findings from Belgium, where a study indicated that most Applicants of international protection (AIPs) have experienced sexual victimization at some point in their lives (84%), and a significant number have experienced it within the preceding year (61%), suggesting an underreporting in this study (16).

Notably, the prevalence of rape (9%) among young migrants is more than twice as high as reported in the national survey (4%), indicating increased vulnerability (22). It also exceeds the weighted incidence of rape (4.8%) reported among recently arrived asylum seekers in France (27). However, it is lower than the prevalence of rape (14.9%) reported by Doctors of the World (*Médecins du Monde, MdM*) in their 2014 report on access to healthcare for vulnerable people, such as migrants, asylum seekers and refugees (MARs), in 11 European countries, where 24.1% of women and 5.4% of men reported being raped (28). Additionally, a UK study found that 76% of refugee and asylum-seeking women had been raped either in their home country or in the United Kingdom (29). Existing literature indicates that rape is the most frequently reported form of SV among migrants. However, and rape rates vary widely across studies and countries (13–15). These variations highlight the diverse patterns of victimization experienced by different migrant populations and underscore the necessity for context-specific research to inform effective interventions and support mechanisms. Differences in rates can be attributed to variations in the definitions of SV, the normalization of its occurrence in some contexts, distinct understandings of victimhood, characteristics of the studied populations, research methods, statistical procedures, and outcome measures (12–15). While our study was conducted in school settings and included all categories of migrants, other studies reporting higher victimization rates have been conducted in health care settings and refugee camps and focused on vulnerable migrant populations, such as refugees, asylum seekers, and undocumented migrants (12, 14).

### 4.2 Disparities in victimization based on age, gender and sexual orientation

Our descriptive findings reveal significant disparities in SV and rape rates based on age, gender, and sexual orientation. Teenagers and youths reported higher rates of SV (26 and 27% respectively) and rape (10%) compared to those aged young adults (21% for SV, 7% for rape), suggesting that younger age increases the risk of victimization. This observed pattern aligns with trends reported in national and international studies (15, 22). Additionally, non-binary individuals experience SV or rape more frequently, with rates of 45% for SV and 17% for rape, compared to men (26% for SV, 10% for rape) and women (21% for SV, 6% for rape). Similarly, those who identify as LGBA report higher prevalence of SV (38%) and rape (14%) than heterosexual individuals (24% for SV, 8% for rape). Similar patterns emerge when comparing transgender (45% for SV, 8% for rape) and cisgender (26% for SV, 4% for rape) individuals in national surveys. Despite the challenge of direct comparison due to separate LGB results for men and women, this trend persists when comparing LGB individuals to heterosexuals (15, 22). Although descriptive, our results suggest a compounded effect of minority gender and sexual orientation status along with migrant status, aligning with findings from previous studies (30, 31).

Existing evidence highlights that both male and female young migrants experience SV, with vulnerabilities stemming from multiple sources across socioecological levels (15). However, our descriptive and bivariate findings challenge conventional patterns observed between men and women in national surveys and most international studies (14, 21, 22). Unlike the prevailing trend where SV rates are higher among women aged 16–29 years, our research reveals a different trend among young migrant men (12, 14, 21, 22). Specifically, young migrant men (26% for SV, 10% for rape) report higher incidences of SV and rape compared to young migrant women (21% for SV, 6% for rape). However, young migrant men report SV at a rate 10 times higher (10%) than young men (1%) in the general population and higher rate than women (7%) in the general population, emphasizing their increased vulnerability (22). Previous research indicates that all genders are vulnerable to SV, although women remain disproportionately impacted (12, 14, 15). Depending on the studied population, some studies show higher prevalence of SV/rape among men compared to women, others show the reverse pattern, and still others report more gender-balanced victimization rates (12, 15). Several studies highlight higher SV rates among men, boys, and LGBTQ+ migrants throughout migration trajectories (30, 31). In specific contexts, such as armed conflict zones, male victims—especially younger males—are at significant risk of SV victimization, sometimes surpassing rates experienced by females and girls (13). Despite increased vulnerability due to intersections of sexual orientation, gender identity/expression, and migrant status, research and interventions targeting male victims and LGBTQ+ individuals remain less prevalent, with most studies primarily focusing on female victims (14, 31, 32).

### 4.3 Determinants of sexual violence and rape victimization

Our findings highlight key determinants of SV and rape victimization among young migrants. Specifically, level of education, gender, sexual orientation, and legal status significantly impact SV victimization. Meanwhile, gender identity, sexual orientation, and the socio-political context in the country of birth are key determinants of rape victimization.

Participants with at least 10 years of education frequently reported SV incidents. Higher education seems to foster better understanding of victimhood, sexual consent, and rights. Highly educated individuals may be more equipped to recognize and address SV, leading to higher reporting rates. Surprisingly, those with 10 years or less of education also frequently reported incidents of rape, raising questions about vulnerability among migrants with low education. The National survey reveal that sexual harassment and other sexual offenses are more common among women with post-secondary education compared to those with pre-secondary education. However, women with pre-secondary education are more frequently subjected to forced sexual intercourse with physical violence or threats (22). In addition, previous research indicates that lower education levels correlate with higher levels of male IPV perpetration and female IPV victimization (33).

Our results confirm the heightened vulnerability to SV and rape victimization faced by non-binary and LGBA young migrants. Existing literature underscores that LGBTQ+ migrants are at extreme risk of various forms of violence, including SV. Their intersecting marginalized identities contribute to this vulnerability (30, 31). Throughout the migration journey, these individuals encounter distinct forms of violence and abuse. These include severe and prolonged incidents related to sexual orientation or gender identity during predeparture. During travel, they continue to face victimization and a high risk of SV. Detainment-and deportation-related violence occur during interception and return. Additionally, new manifestations of violence and abuse emerge while living with past trauma at the destination (30, 31). However, previous studies have primarily focused on gay men, limiting the ability to examine differences in vulnerabilities among migrants with diverse sexual orientations, gender identities, and expressions (31).

The higher prevalence of rape victimization among young migrants born in non-conflict settings confirms that vulnerability to SV persists throughout the migration journey, not only in the country of origin but also during travel, transit, and at the destination. Studies conducted in transit (e.g., Libya) or within destination countries (including European nations) reveal high rates of SV victimization, particularly among female migrants (13). Additionally, a Belgian study revealed that most participants who disclosed sexual victimization within the previous year likely experienced at least one incident in Belgium, as their average stay was nearly 11 months (16). According to another study conducted by MdM, 21.1% of reported rapes and 17.7% of sexual assaults occurred after individuals had arrived in the destination country (28). These figures underscore the critical need to adopt a migration-trajectory approach, (which outlines the distinct phases of the migration journey) when examining sexual victimization in this group. It is imperative to identify factors related to sexual victimization that occur prior to departure, during transit, and following arrival in the destination country. Understanding these aspects is vital for developing effective policy recommendations, support systems and prevention strategies.

The vulnerability of migrants to SV is also influenced by their legal status, bureaucratic processes, and access to support services (13, 15, 34). It is unsurprising that migrants still awaiting a decision on their residence permit face an increased risk of victimization. The lack of a residence permit places them in a vulnerable position. Lengthy bureaucratic processes and national legislation impact the rights of migrants without permits (e.g., irregular migrants, asylum-seekers, and partner visa holders) and can inadvertently increase their vulnerability to gender-based violence (GBV) particularly SV. These processes prolong the time migrants spend in precarious situations (15, 16, 35). Asylum-seekers and partners with provisional statuses often encounter restrictions on legal and social rights. Limited access to information and formal public services can exacerbate marginalization and economic hardship. This heightened vulnerability, in turn, increases susceptibility to sexual exploitation and violence. The inability to provide for oneself legally leaves migrants with illegal income options (e.g., black-market employment, criminality, sex work), further enhancing vulnerability (13, 15, 16, 35). Migrants may also fear that reporting incidents could impact their residence permit or asylum application. Moreover, lack of secure accommodation, especially for asylum seekers, undocumented migrants and unaccompanied minors, makes young migrants vulnerable to SV and exploitation. Homelessness, crowded living conditions, and dependency on others create unsafe environments and contribute to this vulnerability (15, 34, 35). While support services exist, accessing them can be challenging due to complex barriers faced by migrants (24). For instance, in Sweden, while awaiting the decision on their asylum application, asylum seekers can choose to stay with friends or relatives or live in accommodation provided by the Swedish Migration Agency. Young single asylum seekers are typically required to share a room with others of the same sex, often in a flat, while families are always provided with their own room. Unaccompanied children are assigned accommodation at a youth home for asylum seekers or placed with a family (36).

### 4.4 Most perpetrators are strangers to the victims, but intimate partner violence also occurs

Our findings show that approximately 46% of sexual perpetration incidents involved strangers. Interestingly, a similar trend emerged in a previous study among migrants in Belgium, where roughly 60% of sexual victimization incidents were perpetrated by unknown individuals (16). However, an earlier study conducted in Belgium and the Netherlands found that, consistent with findings from studies in the general population, most victims knew their assailants, with only 12% being strangers (37). The observed differences may be attributed to varying contexts and life situations that make migrants vulnerable to different types of perpetrators (strangers or partners) at various stages of the migration process. A review reveals that sexual and gender-based violence (SGBV) can change forms throughout the migration journey as migrants are exposed to different vulnerabilities (13).

Notably, participants from non-conflict areas (79%), teenagers (57%) and non-binary (55%), those who chose not to disclose their sexual orientation (59%) and LGBA (57%), individuals with 9 years of education or less (63%), those who move as family ties (59%) and those who received a residence permit in 2015 or earlier (59%) reported the highest prevalence of SV by strangers. These groups also reported SV incidents to a greater extent, suggesting that these vulnerable populations are more susceptible to victimization by strangers throughout the migration process.

Partners were the most frequently mentioned perpetrators (25%) with a known relationship to the victims followed by family members/friends (16%), colleagues (15%), and others (11%). This pattern holds true across various socio-demographic groups, except for non-binary individuals, who identified colleagues as the second most common perpetrators. More than half of the papers included in a review (10 out of 18) reported the occurrence of SV by intimate partners, with prevalence rates ranging from 4.3 to 30% (14). Our results also suggest that intimate partner violence (IPV) is a prevalent issue among young migrants. However, a Belgian study shows a lower prevalence of common perpetrators with a known relationship to the victims, such as (ex)partners (10%) and family members or friends (6%). In contrast, 27% of victims identified a date as the perpetrator, indicating that loneliness may increase the vulnerability of migrants to SV (16).

Young migrant women and LGBA individuals more frequently mentioned partners as perpetrators compared to men and heterosexual individuals, highlighting their vulnerability to IPV. Previous research indicates that female irregular migrants and asylum-seekers faced heightened risk due to migration-related stressors. These stressors impact the perpetrators (38, 39). GBV may serve as a means to assert or maintain power during economic hardships. Traditional gender roles disrupted by migration could contribute to both IPV and GBV perpetration (40–42). A literature review identified several factors linked to IPV perpetration and victimization among asylum-seeking and refugee populations. Demographic factors such as young age, male sex, and alcohol use were associated with perpetration. Lower education levels correlated with both perpetration and victimization. Female forced sex victimization was linked to legal status while (marital status) divorced, separated, or bereaved individuals were more vulnerable to victimization (33).

Nevertheless, nearly one in ten participants reported incidents involving ‘other’ perpetrators (teachers, bosses, or supervisors) beyond the usual categories (strangers, partners, family members, friends, or colleagues). Evidence indicates that, in some cases, the perpetrators were individuals in positions of authority, including those assigned to protect the migrants, or carers of young migrants, in the case of female perpetrators (15). Besides, qualitative narratives suggest that sexual victimization often occurred within migrant communities, especially in refugee camps. Additionally, human traffickers and locals who offered assistance sometimes exploited migrants (15, 16, 30). Notably, European citizens were identified as assailants in a third of reported incidents (16). Sexual victimization among young migrants is a critical issue, and understanding the dynamics of perpetrators is essential for effective prevention and targeted interventions to support survivors.

### 4.5 Most survivors do not disclose the incident, and reporting to authorities is even less common

Despite the magnitude of the problem, 64% of survivors choose not to disclose incidents, indicating that the current prevalence may be underestimated. Previous research also reveals low disclosure rates of SV, with non-disclosure rates ranging from 33 to 64% (15). These low rates are attributed to factors such as stigma, fear of retaliation, blame, ostracization, lack of social support, lack of trust in professionals, and language barriers (12, 15). Additionally, racism, homophobia, and xenophobia may further discourage migrants from seeking post-SV care (31, 34). A Swedish study involving health professionals reveals that young migrants face numerous challenges, with SV being just one of many and often not the highest priority (35).

When they do disclose, they often turn to trusted individuals such as friends or family (29%) rather than authorities (3%). This pattern mirrors findings from Belgium, where 62% of survivors did not report incidents, primarily confiding in friends (70%) and family (25%) without seeking professional help (16). In contrast, the national survey shows higher reporting rates to friends and relatives (52%) and police (6%), indicating lack of social support and possible barriers for migrants to access support systems (22).

Reporting patterns do not significantly differ between those born in conflict and non-conflict settings, suggesting common barriers related to migrant status, such as cultural and language barriers, cost of care, legal status, lack of information about services, confidentiality concerns, community stigma, and multiple discrimination (13). In Sweden, several factors contribute to the low rates of reporting SV among young migrants. Cultural barriers, including the communicative taboo surrounding SV, stigma, and fear of retaliation, significantly hinder reporting. Many migrants come from societies where discussing sexual violence is taboo, making it difficult for victims to come forward. Additionally, language and legal barriers, along with limited knowledge of available services, Swedish laws, and rights, further complicate the situation for those affected and prevent them from disclosing and seeking support (20, 43). While a majority across all age groups chose not to disclose, teenagers, with a non-disclosure rate of 60%, were more forthcoming compared to youths and young adults, who had non-disclosure rates of 67 and 65%, respectively. Children and young migrants under 18 are entitled to the same healthcare as children resident in Sweden regardless of their legal status, which can facilitate disclosure during medical encounters (44). Additionally, in the wake of the 2015 refugee crisis, Sweden has implemented a range of interventions specifically designed for unaccompanied minors. These initiatives, focused on fostering social integration, may create a supportive environment that promotes and facilitates open communication within this vulnerable group (45).

Non-binary individuals (75%) and those who decline to disclose their sexual orientation (72%) exhibit the highest non-reporting rates, followed by men (65%) and LGBA individuals (64%). Research indicates that available services are predominantly women-oriented, offering limited entry points for male and LGBTQI survivors, and staff are often inadequately trained to support them. Moreover, restrictive legislation, lack of awareness about available services, and negative attitudes further hinder service utilization for LGBTQI migrants (31, 34). Despite protections under Swedish law, young LGBTQI migrants may struggle to access information about their rights and services (30, 31). Discrimination, disbelief, lack of empathy, and humiliating comments from service providers significantly impede their help-seeking behavior (30, 34).

These subgroups also confide less in friends/relatives (17% for non-binary, 19% for unknown sexual orientation, 28% for men and 26% for LGBA individuals) compared to women (38%) and heterosexuals (32%). Similar patterns were observed in the national survey for the same age group, where heterosexual women (72%), bisexual women (75%), and lesbians (88%) disclosed SV more frequently to friends and relatives than heterosexual men (51%), bisexual men (67%), and gay men (41%). However, young migrant men, non-binary individuals, and LGBA migrants in this study disclosed less frequently to friends and relatives than their counterparts in the national survey, highlighting a lack of social support among migrants (22). In addition, none of the non-binary and LGBA individuals reported the incidents to the authorities. Previous research highlights that LGBTQI migrants face violence from family, community members, and officials, making it difficult for them to disclose to these potential perpetrators (30, 31).

Those awaiting decisions on their applications (69%) and those who migrate due to family ties (67%) are the most reticent to disclose incidents and the least likely to report to authorities. Policies restricting asylum seekers’ access to healthcare and legal protection may deter survivors without formal resident status from seeking services (35, 44). In Sweden, asylum seekers and undocumented migrants aged 18 or older are only entitled to care that cannot be postponed, which limits their opportunities to disclose incidents and seek post-violence care. Although they are offered a free health assessment where they can disclose such incidents, concerns about being reported or deported, and the potential impact on their asylum case or residence in asylum facilities can influence reporting behaviors, leading to underreporting of SV (16). Additionally, the attitudes of healthcare staff toward asylum seekers and undocumented migrants can further affect their willingness to report sexual violence, often leading to underreporting (43). Moreover, findings from previous research suggest that women dependent on their partners for legal status often do not report intimate partner violence or domestic violence due to fear of deportation or compromising their access to protection and services (34). In Sweden, spouses, registered partners, and cohabiting partners, as well as future spouses or cohabiting partners from non-EU countries, can receive a temporary residence permit for up to 2 years. To extend this permit, they must remain in the relationship with their partner. Exceptions are made in certain cases, such as if they have ties to Sweden (e.g., a new partner or child) or if they have been abused (46). This temporary residence permit can put individuals in a vulnerable position, increasing their risk of exposure to domestic violence, including sexual violence and other abuses. It also discourages them from reporting such incidents to the authorities due to fear of not being able to extend the permit once the relationship ends. However, this study reveals a reverse pattern for survivors who moved for family ties, with women reporting to authorities more frequently (4.3%) than men, none of whom reported (data not shown). This suggests that migrant men and boys face more barriers to accessing post-violence services, whereas women and girls may have more entry points. Previous research indicates that male survivors are hesitant to seek care at women-oriented service points, such as post-SV care associated with maternity-related services or women’s centers focused on gender-based violence (13, 34). This highlights the need for more inclusive and gender-sensitive support systems.

### 4.6 Methodological considerations

This study uses a relatively large sample of participants who understand one of the six languages used in the survey. Participants with limited literacy were afforded assistance during face-to-face data collection by project assistants fluent in their native language. The survey’s administration in schools and other venues, though post sent home and online facilitated the inclusion of migrants from diverse backgrounds, enhancing participant diversity.

Despite these strengths, some limitations are to be acknowledged. Participants were conveniently selected, possibly excluding individuals due to language barriers and introducing selection bias. While the sample’s gender and region of birth characteristics likely mirror newly arrived migrants in Sweden, generalizing to other migrant groups may be limited. The cross-sectional design does not allow causal inference and recall of SV and rape over the life course pose a risk of recall bias. Social desirability due to the self-reported nature of the questionnaire and privacy concerns in schools might have influenced responses, despite efforts to ensure privacy during data collection. Despite limitations, this study provides valuable information about the prevalence of SV and rape, perpetrators, and reporting patterns among newly arrived young migrants in Sweden.

## 5 Conclusion

This study highlights the prevalence of SV and rape among young migrants in Sweden, identifying key determinants and disparities in victimization, perpetrator profiles, and reporting behaviors. The findings urge policymakers to prioritize targeted interventions, raise awareness, provide comprehensive support services tailored to the diverse needs of migrant groups, various perpetrator types, and the individual, systemic, and structural factors influencing reporting behaviors. These initiatives should adopt a migration-trajectory approach that recognize that migrants may have experienced SV throughout their journey and consider the unique experiences and vulnerabilities of non-binary individuals, LGBA individuals, those with low education levels, and migrants without formal residence status, regardless of their origin.

A multi-faceted approach, including policy reform, community engagement, and comprehensive support services, is essential to address the specific vulnerabilities of migrants to SV. Firstly, laws should be strengthened and policies made inclusive to consider the unique challenges faced by migrants, such as housing policies and economic support for asylum seekers, temporary residence permits for spouses/partners, and requirements to stay in relationships for permit extensions, as well as limited access to services for asylum seekers and undocumented migrants. Secondly, culturally and linguistically adapted information about rights, including protections against SV and available healthcare services, legal aid, and support, should be offered to migrants as soon as they arrive. Thirdly, community engagement through awareness campaigns and the involvement of community leaders is crucial. Awareness campaigns can help raise awareness about SV, consent, and available resources among migrant communities, while engaging community leaders can foster trust, encourage the reporting of SV incidents, and support victims. Fourthly, professional training for healthcare providers, law enforcement, and social workers is necessary to equip them with the skills to recognize the specific vulnerabilities of different migrant groups and respond appropriately to cases of SV against them. Lastly, a multi-stakeholder partnership, including government agencies, non-profits, and migrant organizations, is crucial to coordinate efforts, strengthen reporting mechanisms, and share best practices. Ensuring there are anonymous and safe channels for migrants to report SV without fear of retaliation, deportation, or stigmatization is also vital.

Implementing these strategies can help create a safer and more supportive environment for migrants, reducing their vulnerability to SV and ensuring they have access to the resources and support they need, regardless of their background (conflict setting or not) or legal status. This study underscores the need for further investigation into the experiences of vulnerable subgroups and the barriers influencing the reporting behaviors of survivors within migrant communities.

## Data Availability

The datasets analyzed in this study are not publicly available because of the Swedish legal restrictions on personal data and the current ethical approval for the study, but they can be made accessible upon reasonable request and after obtaining the necessary permissions.
